# Acute generalized exanthematous pustulosis sine pustules: A case report

**DOI:** 10.1016/j.jdcr.2026.02.044

**Published:** 2026-03-16

**Authors:** Aruba Ali, Margaret A. O’Brien, Sylvia Hsu

**Affiliations:** Department of Dermatology, Temple University Lewis Katz School of Medicine, Philadelphia, Pennsylvania

**Keywords:** acute generalized exanthematous pustulosis, AGEP sine pustules, cutaneous adverse drug reaction, drug eruption, hydroxychloroquine

## Introduction

Acute generalized exanthematous pustulosis (AGEP) is a severe cutaneous adverse drug reaction with an estimated incidence of 1-5 cases per million annually.^1^^,^^2^ It is most often triggered by antibiotics, calcium channel blockers, and antimalarials such as hydroxychloroquine. AGEP is typically characterized by an acute onset of sterile pustules on erythematous, edematous skin, accompanied by fever and leukocytosis.

The pathophysiology of AGEP involves a type IVd hypersensitivity reaction mediated by drug-specific T cells, neutrophilic chemotaxis, and cytokine release.^3^ Histopathology demonstrates spongiform subcorneal or intraepidermal pustules, papillary dermal edema, and perivascular infiltration of neutrophils and eosinophils.

Recently, atypical variants have been described, including AGEP sine pustules, in which patients exhibit erythematous eruptions without clinically visible pustules.^4^^,^^5^ Such cases may mimic morbilliform drug reactions or drug reaction with eosinophilia and systemic symptoms (DRESS), complicating diagnosis.^3^^,^^6^ Recognition of this variant is essential, as timely withdrawal of the culprit drug and appropriate management significantly improve outcomes.

We present a case of hydroxychloroquine-induced AGEP sine pustules, contributing to the growing body of literature on this rare clinical entity.

## Case report

A 53-year-old woman presented to our outpatient clinic with a 7-day history of a rapidly progressive, pruritic rash. The eruption began on her back and spread to her trunk and upper and lower extremities within several hours. She presented to her local emergency department where she was diagnosed with drug reaction with eosinophilia and systemic symptoms. Laboratory testing at that time revealed leukocytosis (12.89 × 10^3^/μL), eosinophilia (13%), and an absolute neutrophil count of 8.38 × 10^3^/μL. She was prescribed clobetasol ointment and was discharged with referral to dermatology.

Her medical history included psoriatic arthritis treated with risankizumab for several years. Two weeks before rash onset, she was started on hydroxychloroquine for worsening joint disease. She discontinued hydroxychloroquine 1 day after rash onset.

**Fig 1:**
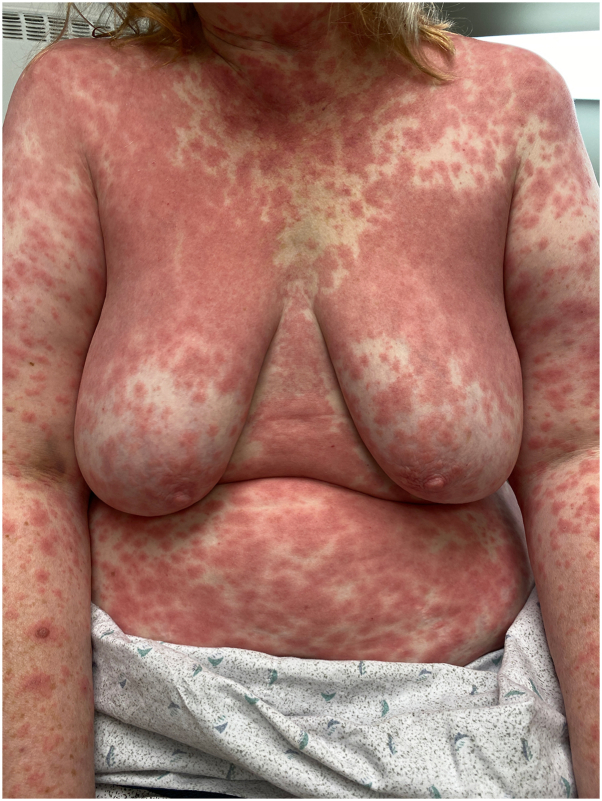
Morbilliform eruption with confluent erythematous patches without clinically evident pustules on the trunk and extremities.

On examination in our clinic, she had widespread erythematous macules coalescing into patches over the trunk (Fig 1) while sparing the folds. Clinically, there were no obvious pustules or facial edema. There was no mucosal involvement or lymphadenopathy. Given the clinical course and culprit medication with a known delayed latency, our clinical diagnosis was AGEP.

**Fig 2:**
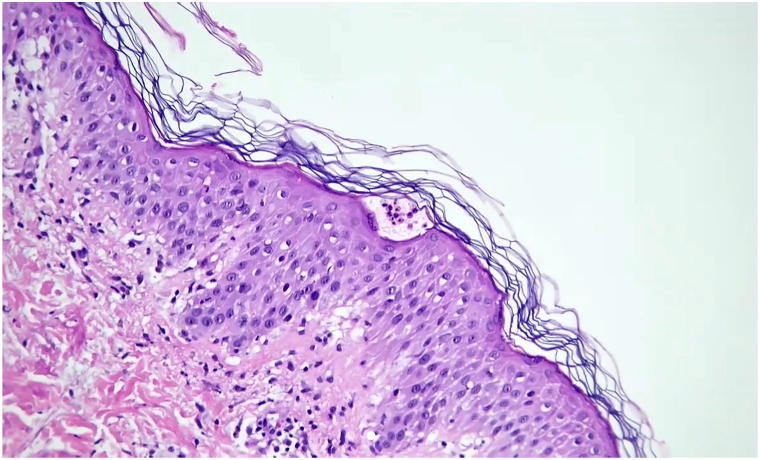
Subcorneal pustules with spongiosis and superficial dermatitis with eosinophils. (H&E 400x)

Subsequent punch biopsy of the left back revealed spongiotic epidermis with microscopic subcorneal pustules (Fig 2) and a superficial and deep perivascular lymphocytic infiltrate with admixed eosinophils and neutrophils. This biopsy was consistent with our clinical diagnosis of AGEP. She was prescribed a prednisone taper (60 mg daily × 5 days, 40 mg × 5 days, 20 mg × 5 days), with complete clearance noted over subsequent follow-up a week later.

## Discussion

AGEP is a rare cutaneous drug reaction, first described in the 1960s, and has been systematically characterized through large case series such as the EuroSCAR study.^2^^,^^7^ Most cases are drug-induced, with antibiotics being the most common triggers, followed by antimalarials and calcium channel blockers.^1^^,^^7^ Hydroxychloroquine, in particular, has been repeatedly implicated in AGEP, often with a longer latency period of up to 2 weeks, as seen in our patient.^1^^,^^6^

While the hallmark of AGEP is the rapid onset of nonfollicular pustules, AGEP sine pustules has emerged as a recognized variant. In 2022, Svoboda et al described several cases of AGEP sine pustules confirmed histologically despite absent clinical pustules.^8^ More recently, Shetty and Veenstra reported semaglutide-induced AGEP sine pustules, underscoring that novel agents can also provoke this phenotype.^4^ These cases, along with ours, highlight that clinicians should not exclude AGEP in the absence of overt pustules. Accordingly, a skin biopsy should be performed in all cases of severe drug hypersensitivity to establish a diagnosis of AGEP sine pustules.

Management consists of immediate discontinuation of the culprit medication, supportive care, and, in moderate to severe cases, systemic corticosteroids.^1^^,^^9^ The prognosis is generally favorable, with most patients recovering within one to 2 weeks.^1^ In our case, a prednisone taper led to rapid clinical improvement.

This case adds to the growing recognition of AGEP sine pustules as a clinically significant variant and underscores the diagnostic importance of histopathology in suspected drug-induced eruptions.

## Conflicts of interest

None disclosed.
